# Comparative evaluation of vegetation indices for water and heat stress detection and monitoring across land cover types

**DOI:** 10.1038/s41598-026-50643-7

**Published:** 2026-04-29

**Authors:** Tibor Zsigmond, Mehjubin Kizhisseri, Ágota Horel

**Affiliations:** 1 Department of Soil Physics and Water Management, Institute for Soil Sciences, HUN-REN Centre for Agricultural Research, Fehérvári St 132–144, H-1116 Budapest, Hungary; 2 Doctoral School of Environmental Sciences, Loránd Eötvös University, Egyetem tér 1–3, H-1053 Budapest, Hungary; 3 CNR-ISMAR Institute of Marine Sciences, Via del Fosso del Cavaliere 100, 00133 Rome, Italy

**Keywords:** Drought tolerance, Soil moisture, Grassland, Cropland, Sunflower, Remote sensing, Vegetation index, Ecology, Ecology, Environmental sciences, Plant sciences

## Abstract

Studying variations in vegetation indices (VIs) under plant stress is still limited by the need for validation across scales, from leaf to field, to reliably distinguish different stressors in agricultural systems. This study aimed to analyze the water and heat stress on sunflower, grass, and forest trees using vegetation indices. We assessed the spatial variations in soil water content, soil temperature, and vegetation indices (NDVI and leaf chlorophyll content) across land use types of cropland (sunflower), grassland, and forest. We used a combination of field measurements and remote sensing data. The research sites are situated close to each other on medium to steep slopes. Soil water content (SWC), soil temperature, VIs of NDVI_F_, leaf chlorophyll content (CCI_F_), and leaf area index (LAI) field measurements were collected at 4 to 5 points along a transect during the vegetation period of 2022. During the study period an extreme drought occurred. Field measurements were implemented using handheld devices. The remote sensing data were obtained from the Sentinel-2 (S2) satellite for NDVI_S2_ or green chlorophyll index (GCI_S2_). Statistical assessment of the relationships was performed using Pearson correlation, PCA, complemented by RMSE and MAE metrics to quantify data precision. Among the land use types, we found distinct differences in SWC with the higest in grassland (15.39%) and the lowest in forest (9.71%; *p* < 0.05); in soil physical and chemical properties (e.g., significantly higher SOC and total N content for the forest and grassland site compared to cropland, *p* < 0.05); and in plant indices . NDVI_F_ and CCI_F_ showed significant differences among all land use types with the highest in cropland (NDVI_F_ = 0.66) or forest (CCI_F_ = 18.8; *p* < 0.05). S2 data detected significantly higher VIs for the forest (NDVI_S2_ = 0.56 and GCI_S2_ = 2.16; *p* < 0.05). However, no significant differences were observed for the VIs within the same land use. NDVI_F_ and NDVI_S2_ only correlated well for grassland (r = 0.83; *p* < 0.001). Our data indicated that LAI is the strongest predictor among the VIs, exhibiting good correlations with CCI_F_, NDVI_F_, NDVI_S2_, and GCI_S2_ (r = 0.71-0.79; *p* < 0.001). The strongest relationships were observed in cropland LAI, with correlation coefficients of r = 0.80 for NDVI_S2_ and r = 0.96 for GCI_S2_.

## Introduction

Changes in meteorological conditions, such as prolonged drought or extreme precipitation events, can have a major effect on agricultural fields and crops, affecting plant development and crop/fruit yield. Climate change related to air and consequently soil temperature rises results in added heat stress to the plants, increase soil evaporation and plant transpiration, and lowers the amount of water available for plants to use. Despite growing recognition of these impacts, a clear research gap remains in quantitatively linking combined stressors, such as heat and water scarcity to the different VIs and to plant responses, across scales.

Vegetation indices (VIs) can be used to monitor any changes in plant response due to environmental parameters. Some of the most widely used VIs are spectral indices of normalized difference vegetation index (NDVI), photochemical reflectance index (PRI), or structural parameters such as leaf area index (LAI). Other plant phsysiological parameters are leaf chlorophyll content and chlorophyll fluoresence, which is a proxy for Fv/Fm, an indicator that allows detection of unfavorable soil and atmospheric moisture conditions or heat stress^1^. However, numerous other plant parameters can be used to monitor plant health, including stomatal conductance or leaf water potential. These vegetation indices have different sensitivities for different plants and crops. The type of plants to be observed is also a major factor in plant responses^2^, such that plant characteristics and consequently their measured parameters shift differently from the regular baseline. Zolin et al.^3^ observed that it takes approximately 5 to 8 days to detect significant changes in NDVI, PRI, and Fv/Fm values in pea and wheat plants, often reaching their minima by day 12 of drought conditions. Zhang et al.^4^ found that chlorophyll content can only be used to estimate water stress in maize under severe drought conditions. However, for vineyard plant measurements, NDVI might be a less useful tool compared to PRI, which is more sensitive to environmental change^5,6^. Mevy et al.^7^ showed significant correlation with the normalized water absorption reflectance (R975/R900) and PRI values, though others found no correlations with PRI and water stress in plants^5^. While calculated VIs can provide robust data on plant health and development, sometimes specific spectral bands can offer a more sensitive response to changes in climatic conditions^8^.

Collecting data on plant health using handheld devices or UAVs can be labor-intensive or expensive; hence, data retrieved from remote sensing (RS) sources can be highly advantageous and relatively inexpensive for stakeholders and research purposes. Some of the VIs can help to improve remote-sensing plant production estimates^9^, can be used for crop assessment, plant stress detection, changes in vegetation status^10^, or for monitoring baseline plant status after extreme weather conditions^11^. Hence, we might be able to better predict vegetation stress solely from remote sensing data after validating our developed model using measured data. However, there are still important factors affecting the final data from RS. The timing of the field measures and RS data is a crucial aspect of validating the usability of RS data for the purpose of reducing or omitting field measurements^12^. Optical satellite data, such as those from Sentinel-2, may be affected by cloud cover at the time of image acquisition^13–15^, or major events (e.g., precipitation or crop management) can occur between field measurements and satellite overpasses, making direct comparisons difficult. RS data should be validated with ground truth measurements so that background noise from less covered vegetation areas can be eliminated; consequently, plant data or leaf size measurements can be more accurate. Because the canopy cover and its characteristics, such as its greenness varying for different plants^16^, it is also advised to get measurements or data specific to a given agricultural site. In areas with bare soil affecting VIs, the soil adjusted VI (SAVI) can be used^17^. However, areas such as forests, grasslands, or vineyards with inter-row vegetation, with a mixture of plant species, further influence the overall RS values^12^, making it even more complicated for the data to be reliable. While there are several studies investigating different VIs and their changes during plant stress^18–20^, there is still a strong need for validation from leaf to field scale to reliably distinguish different stressors in agricultural systems.

The soil-plant-water system is an integrated system in which environmental parameters, such as soil physical and chemical properties, strongly affect the timing of water and heat stress-related signals. Land use types differ in soil chemistry even when in proximity to each other, resulting from processes such as long-term vegetation uptake, annual cycle of organic matter (OM) input and decomposition rates, composition of the OM, different microbial communities, soil management, or land use change^21–23^. Soil physical changes among land use types can result from erosional processes or the presence of binding agents, such as glomalin^24,25^. Soil physical and chemical properties, vegetation types, and their interactions influence soil moisture and the susceptibility of soil temperature to rise, further affecting related processes such as evapotranspiration. Balashov et al.^26^ found strong positive relationships between actual evapotranspiration and soil thermal properties of volumetric heat capacity, thermal conductivity, and thermal diffusivity, which increased with higher soil moisture content. Therefore, significant changes may lead to shifts in VIs when responding to environmental stress conditions, depending on land use type and vegetation present.

The main objective of this study was to evaluate the spatial changes in soil water content and temperature, along with specific vegetation indices of NDVI, leaf chlorophyll values, and LAI within a vegetation period. We ensured that the selected RS indices can be easily obtained and directly measured in the field using handheld devices. However, our primary focus was on water and heat stress on specific vegetation indices of different data sources (field measured and satellite retrieved). This study included measurements from land use types of forest, grassland, and cropland, along their transects, under a continental climate. Besides the general objective, we also investigated the relationships of 1) vegetation and soil parameters, and 2) the satellite-retrieved data with our ground truth measurements to further evaluate how effectively we can estimate plant health using remote sensing. We hypothesized that changes in environmental conditions, such as prolonged drought or changes in air and soil temperature, affect vegetation that is measurable in different vegetation indices.

## Materials and methods

### Site description

**Fig. 1:**
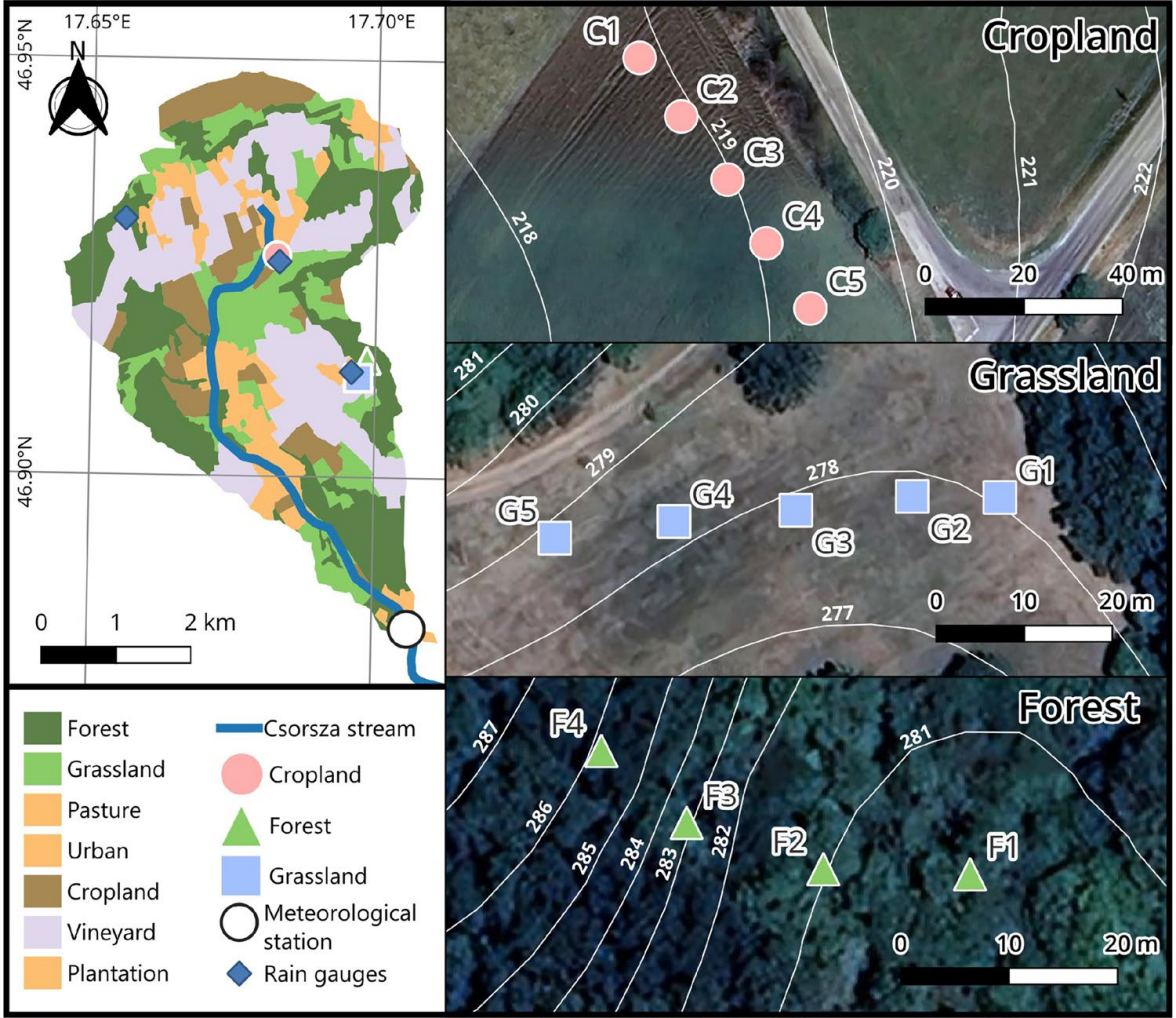
Map of the catchment including the three study sites of cropland, grassland, and forest (CORINE Land Cover^29^; map processing and creation were carried out by the researchers using QGIS^30^ version 3.40.6, https://qgis.org/).

The catchment site is 21 km^2^ and is located in Balaton-Uplands, Hungary. The investigated land use types have areas of 29% forest, 6% grassland, and 13% cropland from the total catchment (Fig. 1). The relatively small size of the catchment allows for detailed monitoring and a clearer interpretation of hydrological responses to land-use patterns, while this site’s structure enable us to investigate the interactions between land use, soil, and plant types on a different scale. The site has a dominantly continental climate with Mediterranean influences, with hot summers and cold winters. The study was carried out in 2022, during the vegetation period (May - October); although some analyses only used data from mature plant growth stages (May - September). The catchment is moderately rain-deficient, with around 578 mm of annual precipitation; however, in 2022, the area experienced a 37% lower annual precipitation (422.8 mm) compared to prior years^27^, resulting in an unusually dry condition.

Generally, the soils at the catchment are Cambisols and Calcisols; however, further classification of the soils showed Epileptic Cambisol (Loamic) for the grassland and Endoleptic Cambisol (Anoloamic) for the forest^28^.

Study slopes had different slope angles for the different land use types (forest 10-15%, grassland ~10%, and cropland < 5%). As we investigated the plant parameters and the effects of soil redistribution processes, each slope was divided into lower and upper portions, while data were collected between these points for further analyses of plant response to environmental changes (Fig. 1).

### Meteorological conditions during the study period

**Fig. 2:**
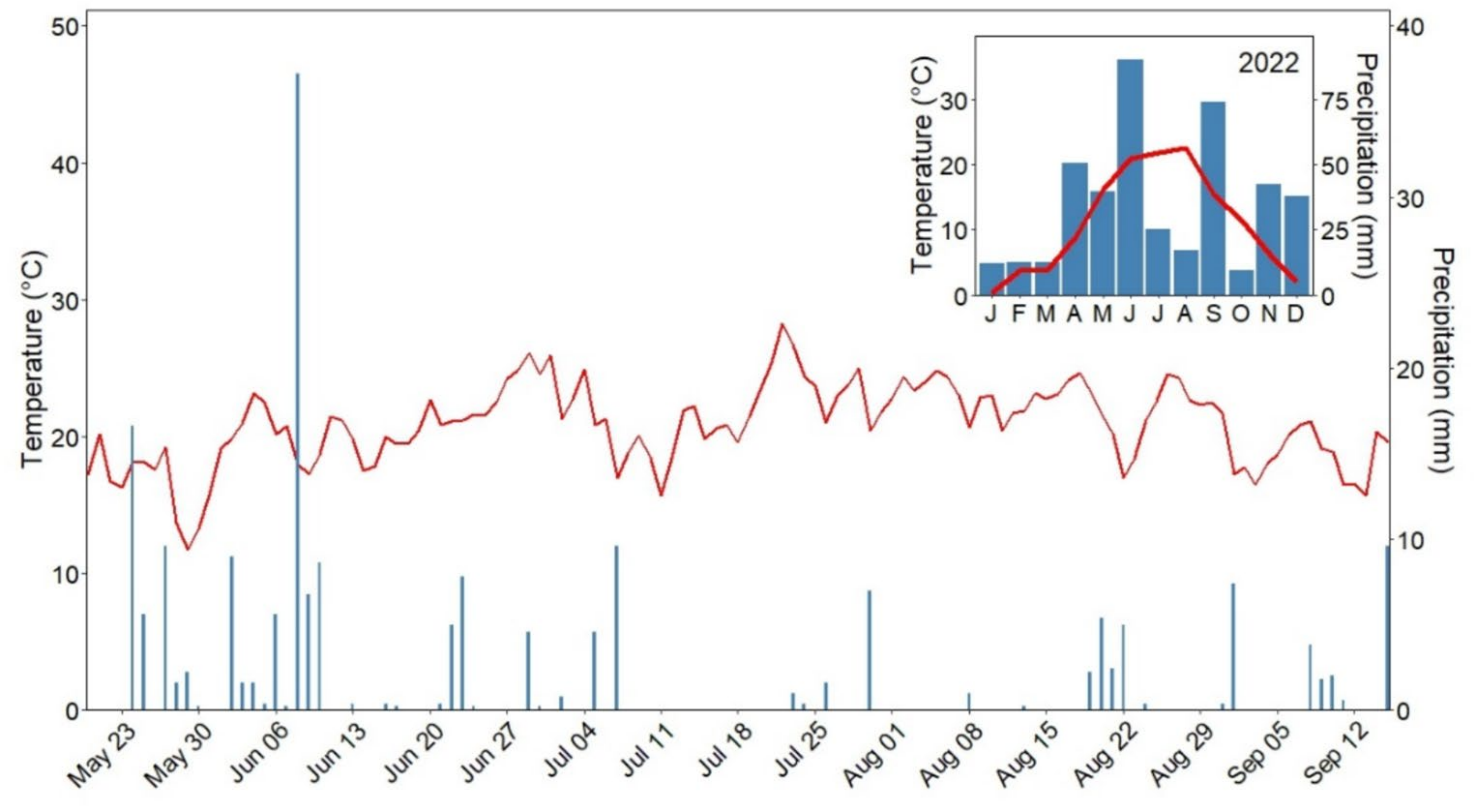
Monthly rainfall sums (blue bars; mm) and average air temperature (red line; Celsius) during the study period.

The meteorological conditions during the study period are shown in Fig. 2. The largest precipitation sums occurred in late May and early June, after which a prolonged rainfall-deficient period or only smaller rainfall events were observed (Fig. 2). Therefore, a longer drought occurred at the catchment during the measuring period, enabling the study of the water and heat stress on VIs.

To determine the severity of drought for our site, we calculated the Selyaninov hydrothermal coefficient (HTC) based on equation 1. Moisture conditions were classified using the HTC according to the thresholds reported in Bartold et al^31^, and the conditions are summarized in Table 1.

**Table 1:** Selyaninov’s hydrothermal coefficients (HTC) during the vegetation period in 2022 according to the meteorological station in Zánka.

**Date**	**HTC coefficient**	**Condition**
May	0.785	very dry
June	1.434	optimum
July	0.368	extremely dry
August	0.244	extremely dry
September	1.597	optimum
July 1 - August 31	0.305	extremely dry
May 1 - September 30	0.689	very dry

1 $$HTC= \frac{\sum_{i=1}^{n}P}{0.1 \times \sum_{i=1}^{n}T}$$

Where P is the total precipitation amount of the given time period n (mm), and T is the mean daily air temperature above 10℃.

### Soil sampling and analyses

We collected triplicate soil samples from the top 20 cm of the soil surface from all upper and lower points. In the present study subsample soil data are not included. Samples were collected once during the study, as previous data from the same sites indcated no significant or temperoral changes in soil physical and most chemical parameters within a one-year period^23,27^; hence, we assumed no further sampling events were required during our study. After air drying and sieving (< 2mm sieve), the samples were analyzed for soil texture and total nitrogen, NH_4_^+^-N, NO_3_^-^-N, soil organic carbon (SOC) content, and pH_H2O_. The amount of total nitrogen was determined using the modified Kjeldahl method (ISO 11261:1995). The amount of SOC was measured based on the Tyurin method by wet digestion^32^. Potassium (K) from K_2_O and phosphorus (P) from P_2_O_5_ were calculated from measurements conducted using ICP-OES (Ultima 2, Thermo Fischer Scientific, Waltham, MA, USA) after ammonium lactate (Al) extraction^33^. The soil pH was measured using a MultiLine P4 (WTW Multi 350i) pH and electrical conductivity meter in 1:2.5 soil-to-water suspensions. Grain size distribution was measured using the sieve-pipette method^34^. Soil element concentrations are reported as mg kg^-1^ dry weight soil.

During the data collection days, we measured SWC for the upper 0-12 cm of soil using a Hydrosense II handheld device (Campbell Scientific) and soil temperature for the upper 3 cm of soil using a digital soil thermometer (Grasstec Group), in three replicates per measurement point in accordance with the instruments’ user manuals.

### Plant measurements - field collected data

The plant measurements started at the beginning of the vegetation period, approximately in May. Each point consisted of around two-three meters in radius. All points’ midpoints were marked; therefore, we used the same locations at each measuring time; however, plants were not marked and chosen randomly at each occasion.

We determined the Leaf Area Index (LAI) of all three land use types (cropland, grassland, and forest) using an AccuPAR LP 80 (Meter Group) instrument, which computes LAI from above and below canopy readings of PAR and leaf angle distribution parameters. LAI measurements were taken only at the upper and lower points of the given slope, where the plant canopy was measured with 40 replicates and averaged.

Different plant indices, such as the chlorophyll content and NDVI_F_ of the plant leaves, were measured from May (chlorophyll) or June (NDVI_F_) to October. For chlorophyll content measurements, we used an Apogee MC-100 instrument, gathering Chlorophyll Content Index (CCI_F_) values for general measurements, with resolution of 0.1 CCI. The instrument measurement area per sample was 63.6 mm^2^. Based on the instrument user manual, the instrument calculates the chlorophyll content from the ratio of optical transmission at 931 nm (NIR wavelength) to the optical transmission at 653 nm (red wavelength), additionally accounting for leaf thickness (equation 2).

2 $$CCI= \frac{\% Transmittance at 931nm}{\% Transmittance at 653nm}$$

At all 14 study points, 15-20 replicate CCI measurements were taken and averaged before analysis. The chlorophyll measurements were taken randomly from the different plant leaves at different heights within the canopy.

Plant leaf NDVI_F_ was measured using a PlantPen model NDVI 310 (Photon Systems Instruments), which, according to the manufacturer, compares reflected light at two distinct wavelengths of 635 and 760 nm. The NDVI values were calculated based on radiance using the following equation^35^:

3 $$NDVI= \frac{Nr/Ni 760\mathrm{n}\mathrm{m} - Nr/Ni 635\mathrm{n}\mathrm{m}}{Nr/Ni 760\mathrm{n}\mathrm{m} + Nr/Ni 635\mathrm{n}\mathrm{m}}$$

Handheld devices for leaf NDVI and chlorophyll measures might become more difficult under extreme drought conditions, and to avoid the resulting data to be less representative of canopy-level properties we ensured high number of replicate measures. Hence, we also measured approximately 15 to 20 replicates per point within a three-meter radius from the mid-point and averaged prior to analysis. All 14 study points of the land uses were measured for NDVI as well. The NDVI_F_ measurements were taken randomly from the different plant leaves at different heights within the canopy.

The PRI values were calculated based on Gamon^35^ using the following equation:

4 $$PRI= \frac{Pr/Pi 532\mathrm{n}\mathrm{m} - Pr/Pi 570\mathrm{n}\mathrm{m}}{Pr/Pi 532\mathrm{n}\mathrm{m} + Pr/Pi 570\mathrm{n}\mathrm{m}}$$

where Pr represents the field stop lens sensor (Meter Group) for reflected radiation from the canopy, while Pi is the hemispherical sensor (Meter Group) for incident radiation values. The reflectance values (Pr/Pi) were calculated for each waveband (532 and 570 nm) and used to calculate PRI.

Since the used devices have different spatial resolutions, we applied systematic repetitions during the measurements. This helped to minimize the scale gaps between the sampling methods and allowed us to reliably extend the point-based data to the studied areas. The direct comparability between satellite data and field measurements relied mainly on NDVI and chlorophyll content, additional measurement points were evenly distributed between the lower and upper sections of the transects.

### Remote sensing of vegetation indices - Sentinel-2 imagery and pre-processing

The remote sensing data were obtained from the European Space Agency’s Sentinel-2 satellite. Satellite data collection and analysis were performed using the Google Earth Engine (GEE) environment^36^. Atmospherically corrected and orthorectified Level-2A (MSIL2A) surface reflectance data were used for the evaluation^37^. The images feature a 10m resolution and a 5-day revisit period. Four images per satellite pass covered our study sites, and the pixel values were averaged. Six satellite passes from May to October 2022 were evaluated to align with the timing of field measurements. Due to the rapid fluctuations in environmental conditions, only those data pairs were included in the comparison where the time gap between field measurements and satellite data acquisition did not exceed one day. This strict temporal synchronization ensures that the observed differences are attributable to sensor characteristics rather than changes in vegetation status. The NDVI values were calculated from the Sentinel-2 B8 (NIR) and B4 (Red) bands using the formula NDVI = (NIR−Red)/(NIR+Red), while the GCI was derived from the B8 (NIR) and B3 (Green) bands using the following equation: GCI = (NIR/Green) – 1^38^. For the analysis, we used the pixel values representing the locations of our field measurement points.

### Statistical analysis

The effects of slope positions and land use types (cropland, grassland, and forest) on soil water contents and plant parameters (leaf chlorophyll content, NDVI, and LAI) were analyzed using nonparametric statistical analyses of the Wilcoxon test and Kruskal–Wallis for the non-normally distributed datasets. Pearson’s correlation coefficient (r) was used to evaluate the linear correlation between the soils’ physical and chemical characteristics and plant parameters. To account for the different scales of the variables, the data were standardized prior to the analysis. Principal Component Analysis (PCA) multivariate analysis was applied to explore the factor pattern of the selected soil and plant parameters. For evaluating model accuracies, we calculated Root Mean Squared Error (RMSE) along with Mean Absolute Error (MAE). The datasets used for the statistical analyses represent the total available data from the three study areas. The specific sample sizes are indicated as n values in the respective table headings and figure captions. All statistical calculations were performed using the software package R (R Core Team, Version 4.0.2). Statistical significance of the data sets was determined at *p* < 0.05.

## Results

### Soil chemical and physical parameters

The soil physical and chemical data from the different land use sites and their two furthest points (hereafter lower and upper points) are shown in Table 2. There were significant differences among the soil chemical and physical parameters within and between land use types. The highest total nitrogen and carbon content was measured at the forest and the lowest at the cropland site (*p* < 0.05). The highest soil pH was observed at the cropland, while the lowest was at the forest site (*p* < 0.05). Based on the texture, the forest soils were silt loam (lower point) or loam (upper point), the cropland soils were silty clay loam (lower point) or silty clay (upper point), and the cropland soils were either clay loam (lower point) or silty clay loam (upper point).

**Table 2:** Physical and chemical characteristics of the soils collected at different slope positions (upper and lower). Sand 2-0.05 mm, silt 0.05-0.002 mm, clay <0.002 mm particle sizes. SOC represents soil organic carbon values. Different letters indicate statistically significant differences at *p* < 0.05. n=18; ±SD.

	**Forest**				**Grassland**				**Cropland**			
	**Upper**		**Lower**		**Upper**		**Lower**		**Upper**		**Lower**	
Total N %	1.2±0.1	a	0.6±0.1	c	0.9±0.1	b	0.4±0	d	0.2±0	e	0.2±0	e
NH_4_-N mg/kg	22.6±5.1	a	12.5±2.8	bc	19.5±3.5	ab	13.8±4	bc	11±1.7	c	7.7±4.4	d
NO_3_-N mg/kg	143.1±41.8	a	69.7±13.9	b	58.8±8.7	b	14.9±2.4	c	11.4±4	c	13.3±10.4	c
SOC %	12.2±1.2	a	6.1±1.7	bc	7.5±0.8	b	5±0.9	c	2.2±0.1	d	1.3±0.2	e
C/N	10.4±0.8	a	9.9±1	a	8.3±0.5	bc	11.4±2.8	a	8.9±0.1	b	8.2±1.1	c
AlK_2_O mg/kg	631±160	a	186±10	d	678±48	a	411±54	b	755±116	a	364±65	c
AlP_2_O_5_ mg/kg	197±47	c	66.4±17.8	d	176±27	c	41.2±2.6	e	424±21	a	395±22	b
pH(H_2_O)	6.8±0.3	c	6.4±0.6	c	7.3±0.1	b	6.7±0.2	c	7.6±0.1	a	7.7±0.2	a
Sand (%)	23.6±1.6	b	19.1±3.6	b	29.6±2	a	20.4±1.9	b	11.5±0.7	d	32.2±1	c
Silt (%)	52.7±2.4	a	57.5±6.3	a	47±1.3	b	43.3±0.8	c	43.4±0.9	c	56.5±0.9	a
Clay (%)	23.7±0.9	c	23.4±3.5	c	23.4±0.7	c	36.3±2.1	b	45.1±0.3	a	11.3±0.7	b

### Soil water content and temperature changes

**Fig. 3:**
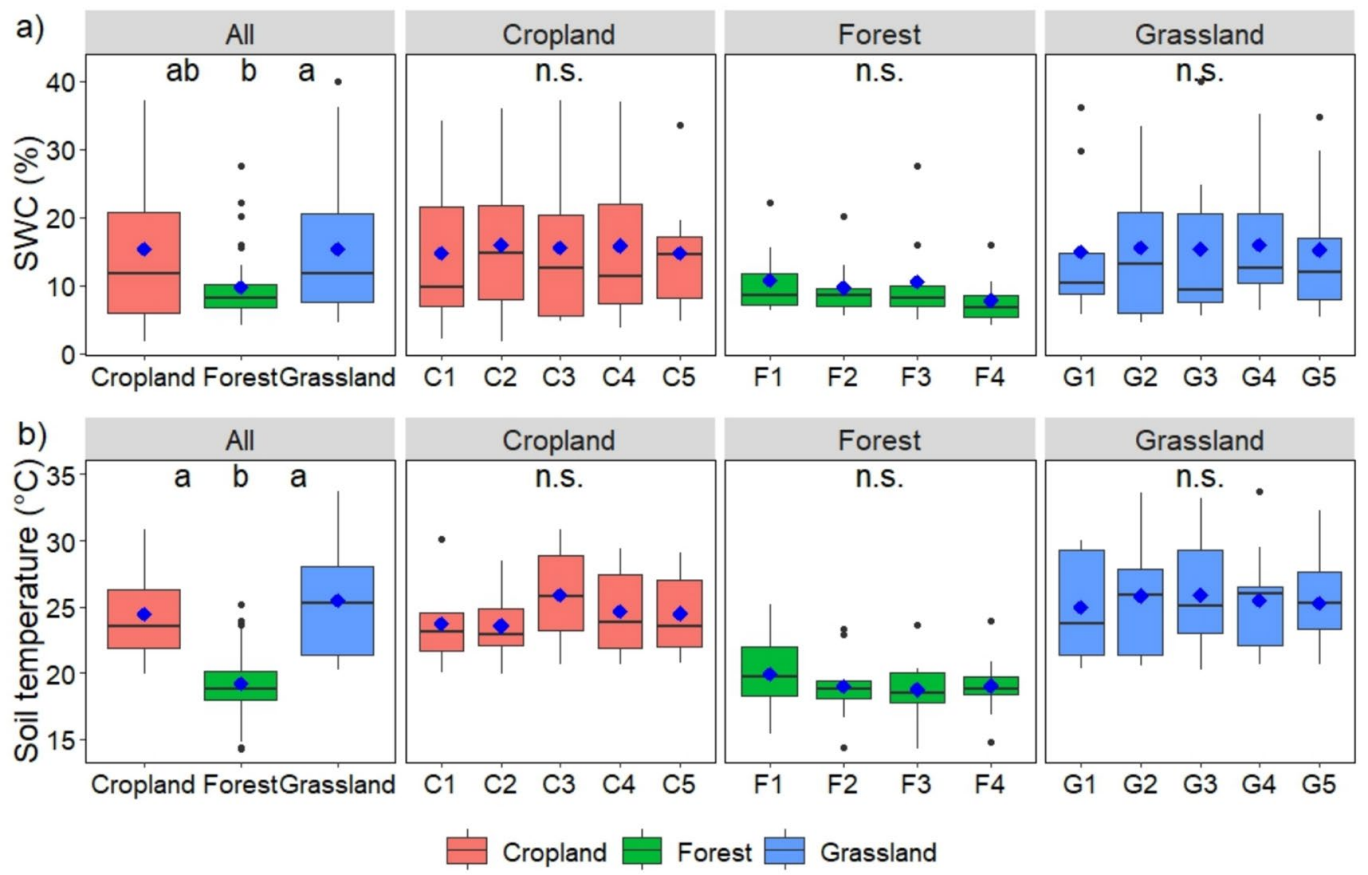
Average (**a**) soil water content (SWC; n=111) and (**b**) soil temperature (n=111) of the three land use types. Each data point represents the averages (diamond), median (solid black line), the mean (red square), the upper and lower quartiles, and the minimum and maximum values (whiskers; data ±1.5 interquartile range).

Among the three land use types, grassland showed the highest soil water content for the upper 12 cm of the soil layer (Fig. 3a). Forest showed significantly lower average SWC compared to the grassland (*p* < 0.05), while the cropland data did not show significant differences. Around September, several larger rain events were occurring at our research site; therefore, noticeable increases in SWC were observed at all sites. While grassland and cropland data showed low variabilities among measurement points, forest showed a noticeable drop toward the upper portion of the slope; however, it was statistically still not significant (*p* > 0.05; Fig. 3a).

A similar, but more pronounced trend was observed between the land use types’ soil temperature data. The forest site exhibited significantly lower overall soil temperatures than the cropland and grassland sites (Fig. 3b), while no statistically significant differences were observed among measurement points within the same land use (*p* > 0.05).

### Changes in plant indices

**Fig. 4:**
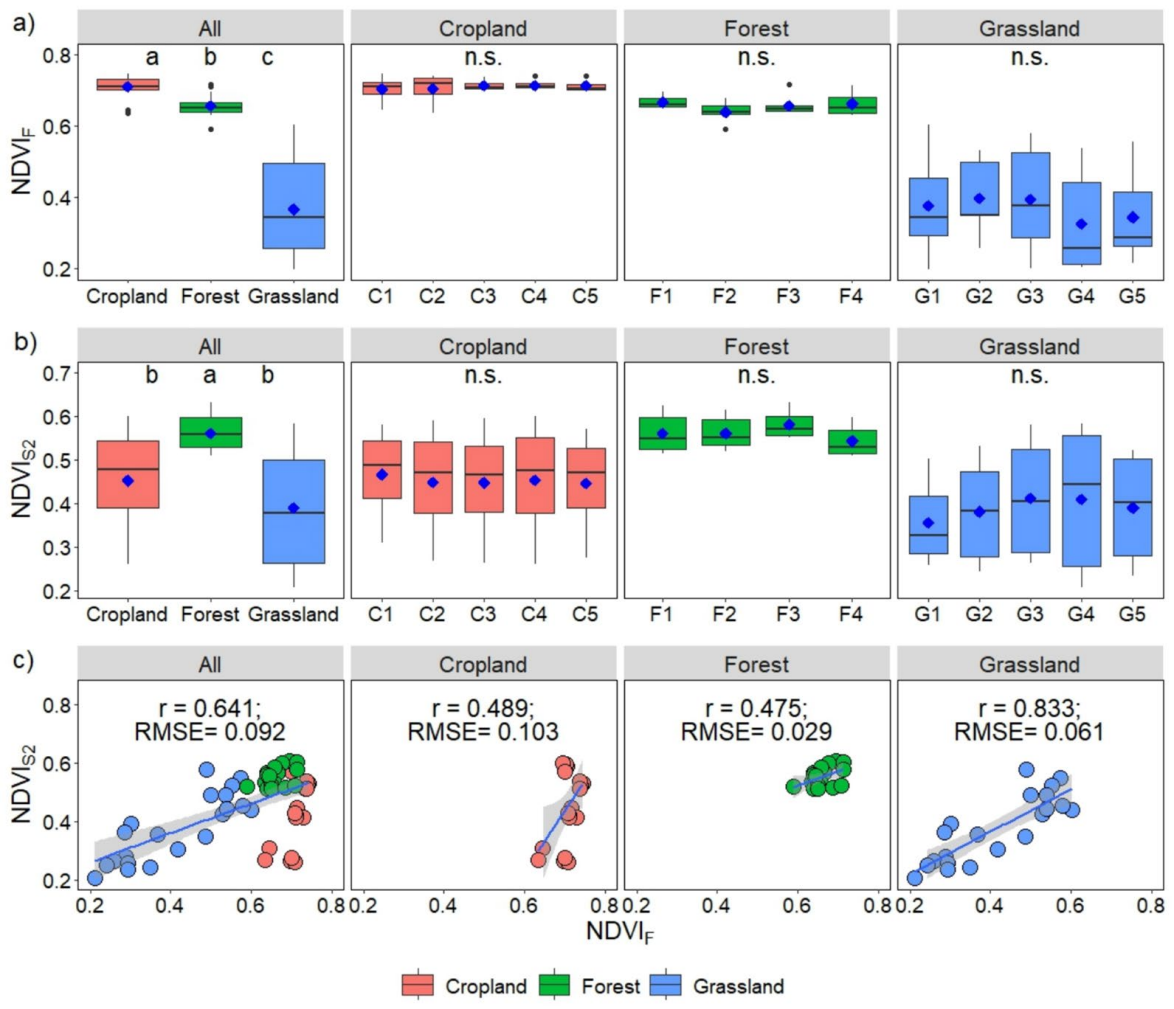
Average NDVI measured for the three land use types using (**a**) field measurements (F; n=77) or (**b**) Sentinel 2 (S2; n=74), and (**c**) the comparison of the different NDVI sources (n=74). Each data point represents the average (blue diamond), median (solid black line), the mean (red square), the upper and lower quartiles, and the minimum and maximum values (whiskers; data ±1.5 interquartile range).

Based on the data collected with a handheld instrument, cropland (sunflower) had the highest mean NDVI value, followed by forest and grassland, respectively. During the time when precipitation values were low, grassland showed the highest changes in NDVI values (Fig. 4a), reflecting increased vegetation stress and loss of greenness. The main vegetation period of the sunflower prior to plant function decline due to senescence (till mid-August), showed an average NDVI of 0.71, while the forest had 0.66, and the grassland 0.42. Grassland also showed the most variation in NDVI values, which increased after the fall rain events. We did not find any significant differences among the measurement points within the same land use site (Fig. 4a).

NDVI_S2_ values were significantly higher for forest compared to the other sites (Fig. 4b), outlining the differences in data collection methods. Both cropland and forest data showed lower NDVI_S2_ values than field measurements, whereas the ranges of NDVI values were higher. S2 data also include background data, such as ground or drier plants, which can be over-represented in croplands or under-represented in forests. There is also a time of data that can distort the results. We found the highest field and S2 data correlation with grassland (r = 0.833; RMSE = 0.061; MAE = 0.05), while cropland and forest data showed moderate correlation among NDVI data sources (Fig. 4c). However, cropland and grassland data were within short value ranges; therefore, their evaluation is limited.

PRI measurements were obtained for the grassland and sunflower plots only, as our insturments could not measure data for the forest vegetation. Consequently, PRI data were used only for general interpretative purposes in this study. Sunflower plants exhibited consistently higher PRI values than the grassland vegetation. Following the pause in rainfall events after mid-June, PRI values declined at both sites, indicating increased water stress.

**Fig. 5:**
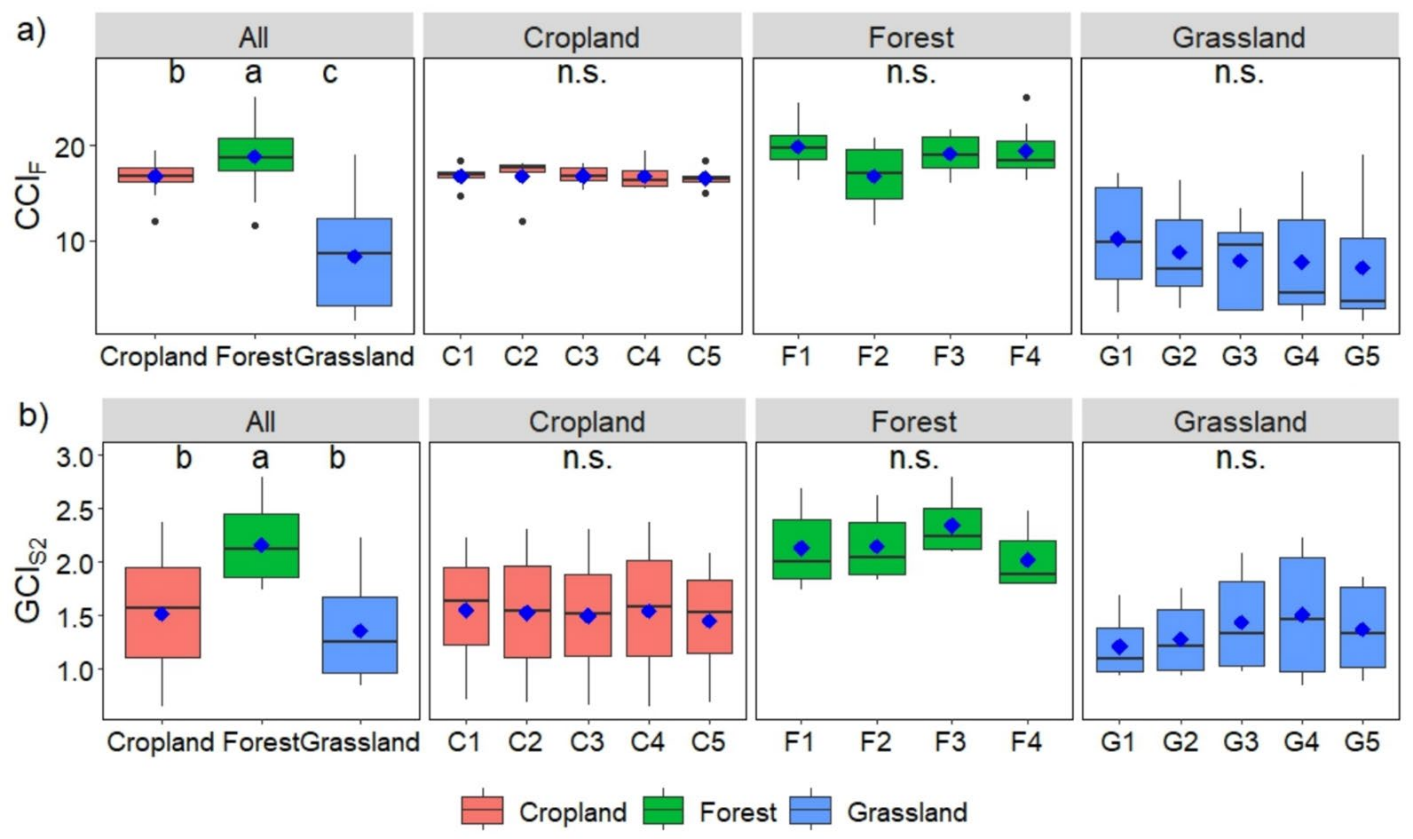
Average leaf chlorophyll content in the three land use types at the different measurement points along the transects measured using (**a**) a field sampling (CCI_F_; n=99), and (**b**) green chlorophyll index (GCI_S2_) retrieved from Sentinel-2 (n=74). Each data point represents the average (blue diamond), median (solid black line), the mean (red square), the upper and lower quartiles, and the minimum and maximum values (whiskers; data ±1.5 interquartile range).

We observed a different trend for leaf chlorophyll values (CCI_F_) among the overall land use types’ data. The highest average values were observed in the forest, followed by grassland, with a high level of data variation (Fig. 5a). The highest CCI_F_ was shown in the June-July period in all three land use types, prior to the point at which the meteorological conditions became extremely dry (data not shown). Similar to the NDVI values, after some rain events during fall, the plants in the grassland received enough water from the soil to revive necessary growth functions, consequently increasing leaf chlorophyll contents that allowed the plants to absorb sunlight, and transfer the necessary energy-storing molecules to perform photosynthesis. Therefore, grassland leaf data increased from 2.11 to 3.91 CCI_F_ from late September to late October, at times when sunflowers were already harvested.

GCI_S2_ calculated from Sentinel-2 data has different unit values compared to CCI_F_; however, it can be comparable when identifying specific trends. We found that, like NDVI_S2_ values, S2 data also showed the highest GCI_S2_ for forest, while cropland and grassland showed no significant correlations (Fig. 5b). When S2 and field data were compared, we found either no correlations between the data or high correlations (i.e., grassland); however, there were no clear trends among the different land use types’ data (not shown).

**Fig. 6:**
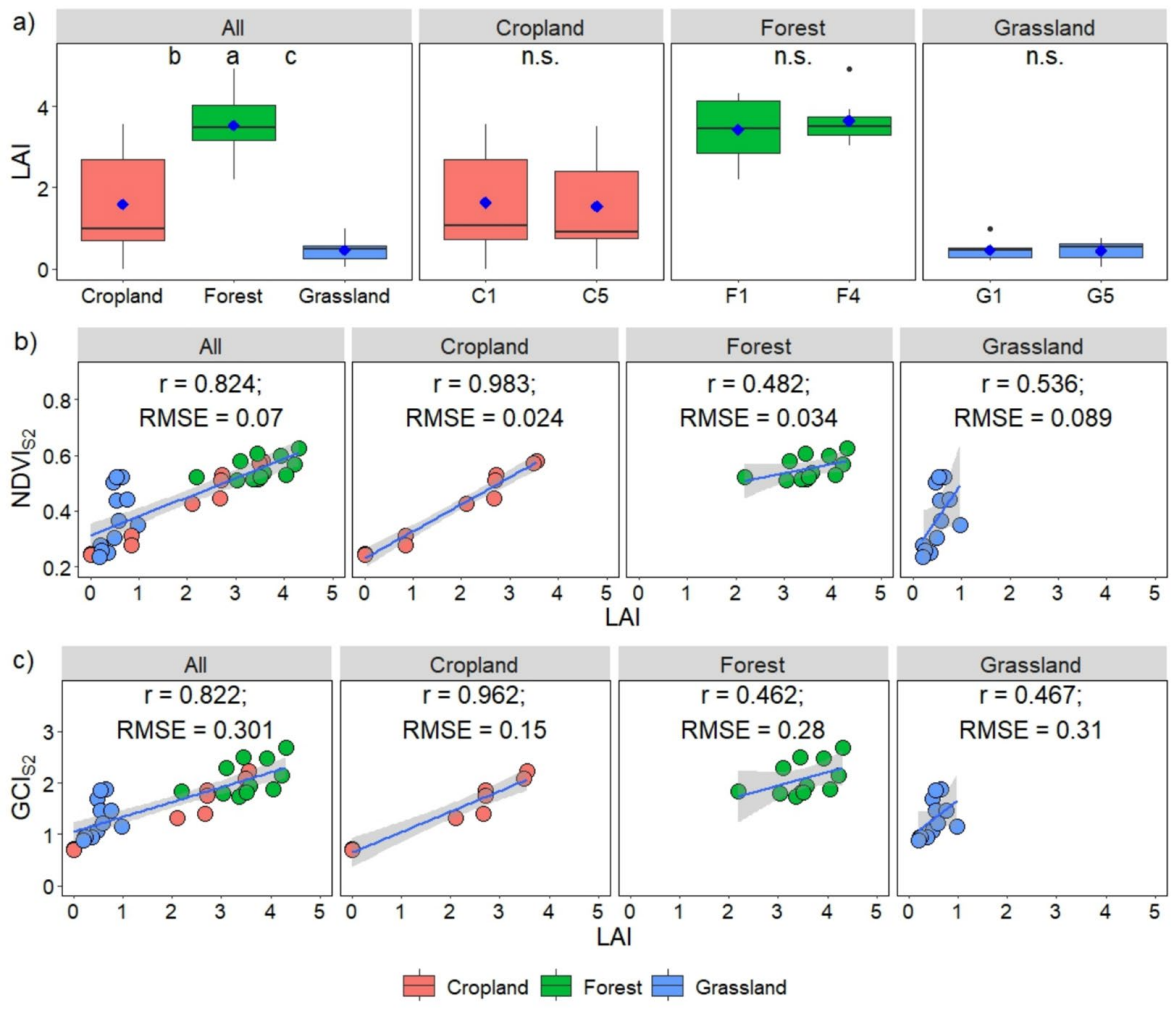
Average leaf area index (LAI) values of (**a**) the three land use types and the upper and lower points of the different land use types (n=42); connections between (**b**) LAI and NDVI_S2_ (n=42), and (**c**) LAI and GCI_S2_ (n=42). C1-C5 - cropland, F1-F4 - forest, G1-G5 - grassland, upper and lower points.

LAI was measured at the upper and lower points of the study transect lines. Grassland showed the lowest, while the forest had the highest mean value for LAI (Fig. 6a). While significant differences in LAI were observed among land use types, no significant differences were found between the upper and lower points. We found strong correlations between NDVI_S2_ or GCI_S2_ and LAI values, with cropland showing the strongest connections (r = 0.976; RMSE = 0.024; MAE = 0.018 for NDVI_S2_ and r = 0.962; RMSE = 0.15; MAE = 0.12 for GCI_S2_; Figs. 6b and c, respectively).

### Correlations between vegetation indices and environmental parameters

**Fig. 7:**
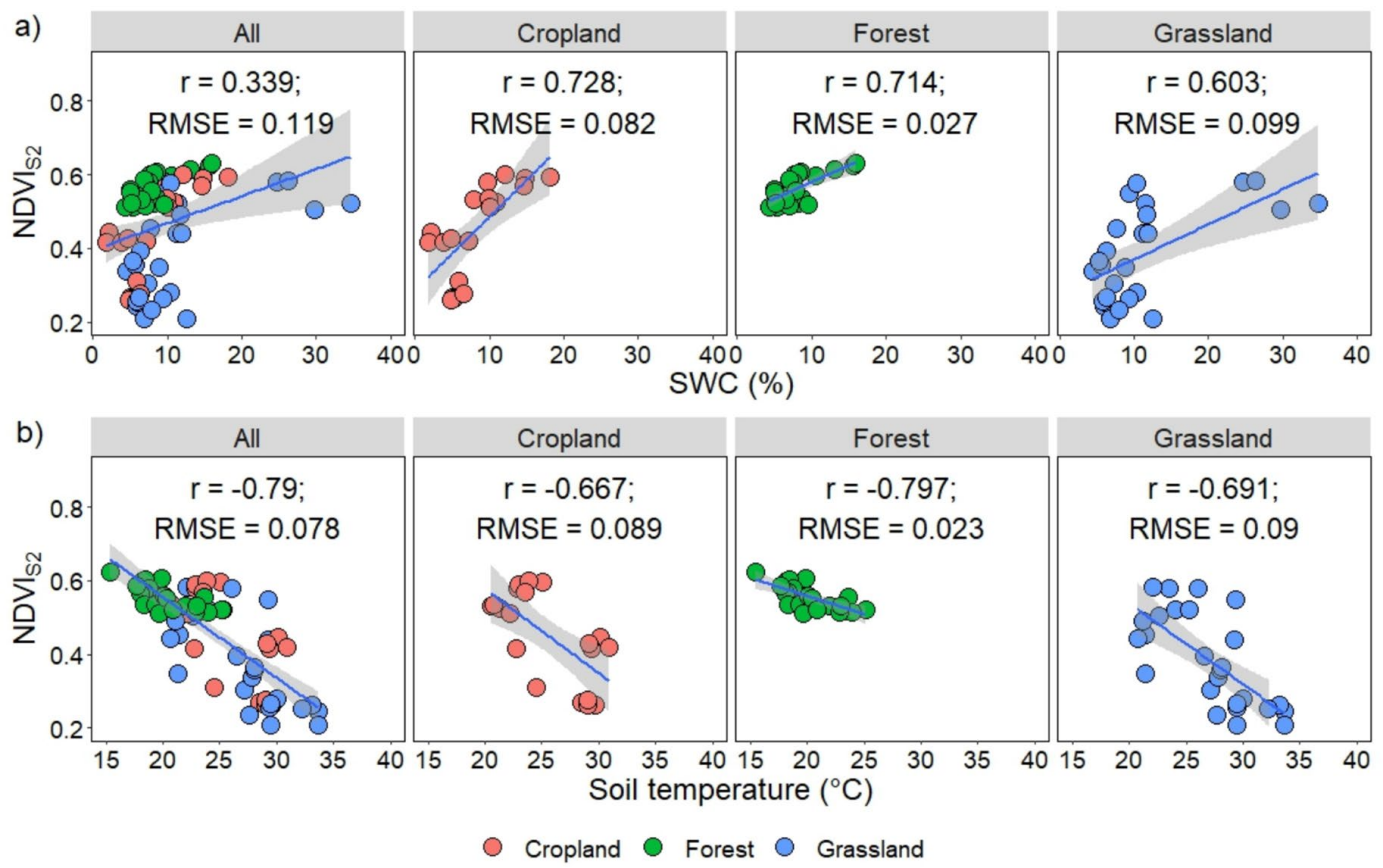
Linear correlation among land use NDVI_S2_ values with (**a**) soil moisture (SWC; n = 69) and (**b**) soil temperature (n = 69)

One of our main aims was to investigate different environmental and vegetation parameter connections when the overall data of all land use types were studied, concurrent with land uses examined separately. Table 3 depicts the overall relationships among the investigated soil and vegetation indices. We found weak positive connections among soil moisture and NDVI_S2_ values (r = 0.339; RMSE = 0.119; MAE = 0.103; Table 3); however, the land uses studied separately showed stronger connections (r = 0.728; RMSE = 0.082; MAE = 0.07 for cropland and r = 0.714; RMSE = 0.027; MAE = 0.02 for forest; Fig. 7a). For NDVI_S2_ and soil moisture, all land uses showed a distinct, separate connection to S2 data.

**Table 3:** Correlation coeffients and Statistical evaluation of the different vegetation and soil indices from field (F) measures and Sentinel-2 (S2) data. SWC refers to soil moisture content, LAI to leaf area index.

	**Site**	**r**	**RMSE**	**MAE**
NDVI_S2_ - NDVI_F_	All	0.641	0.078	0.063
NDVI_S2_ - NDVI_F_	Cropland	0.489	0.078	0.063
NDVI_S2_ - NDVI_F_	Forest	0.475	0.023	0.018
NDVI_S2_ - NDVI_F_	Grassland	0.833	0.090	0.070
NDVI_S2_ - LAI	All	0.824	0.070	0.057
NDVI_S2_ - LAI	Cropland	0.983	0.024	0.018
NDVI_S2_ - LAI	Forest	0.482	0.034	0.032
NDVI_S2_ - LAI	Grassland	0.536	0.089	0.075
NDVI_S2_ - SWC	All	0.339	0.119	0.103
NDVI_S2_ - SWC	Cropland	0.728	0.082	0.070
NDVI_S2_ - SWC	Forest	0.714	0.027	0.022
NDVI_S2_ - SWC	Grassland	0.603	0.099	0.086
NDVI_S2_ - Soil temperature	All	-0.790	0.078	0.063
NDVI_S2_ - Soil temperature	Cropland	-0.667	0.089	0.081
NDVI_S2_ - Soil temperature	Forest	-0.797	0.023	0.018
NDVI_S2_ - Soil temperature	Grassland	-0.691	0.090	0.070
GCI_S2_ - LAI	All	0.822	0.301	0.257
GCI_S2_ - LAI	Cropland	0.962	0.150	0.120
GCI_S2_ - LAI	Forest	0.462	0.280	0.260
GCI_S2_ - LAI	Grassland	0.467	0.310	0.262

For soil temperature, we found a much stronger connection to NDVI_S2_ data, with a negative correlation (r = -0.790; RMSE = 0.078; MAE = 0.06; Fig. 7b). Separately analyzing NDVI_S2_ data for different land use types in relation to soil temperature showed moderate to strong correlations, with forest showing the highest correlation (r = -0.797; RMSE = 0.023; MAE = 0.018; *p* < 0.05). Forest measurements had the smallest range of NDVI_S2_ data collected, while grassland showed a larger range, improving the reliability of our data.

**Fig. 8:**
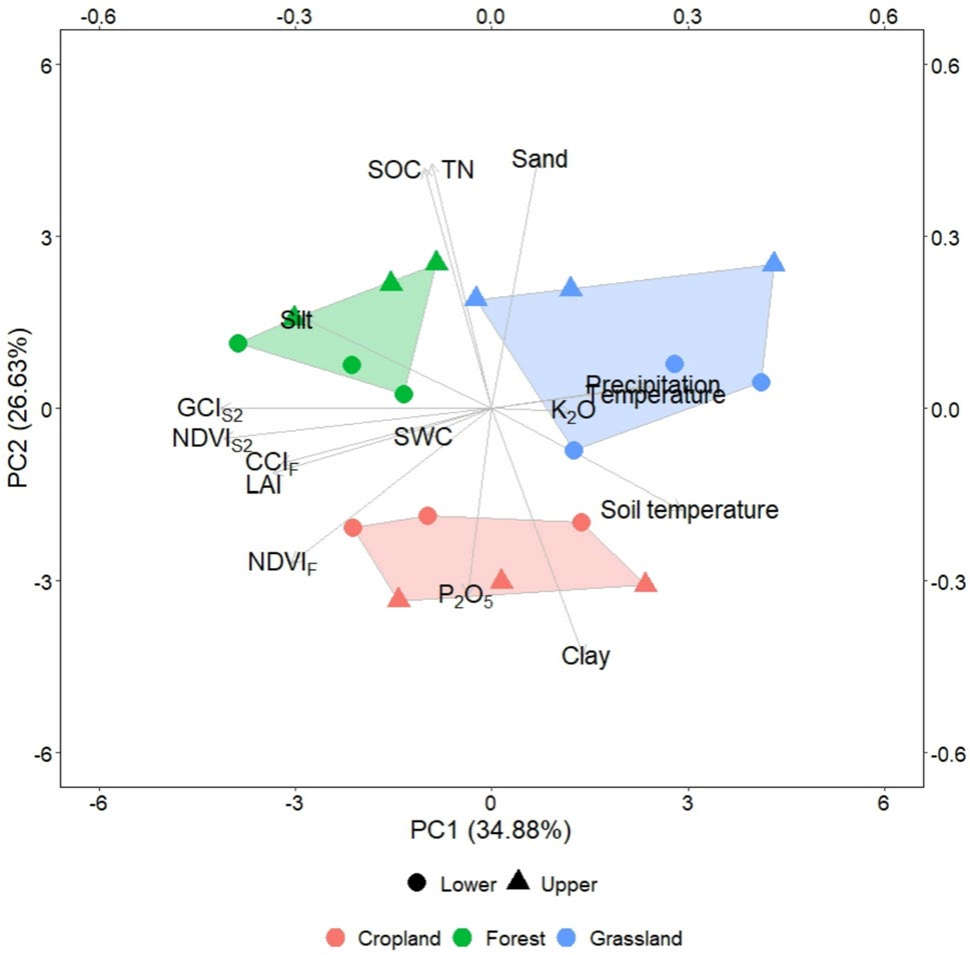
Principal component analysis (PCA) ordination biplot for the three land use types and upper and lower monitoring points with environmental variables (selected soil parameters and plant traits) represented as vectors, and the pairwise representation of the study sites. SWC: soil water content; LAI: leaf area index; SOC: soil organic carbon content; TN: total nitrogen content. S2: data retrieved from Sentinel-2, F: field-collected data (n = 18).

The PCA analyses revealed that the first principal component (PC1) accounted for 37.02% of the variation caused by the interaction, while PC2 accounted for 23.57% (Fig. 8). The data shows clear partitioning of the different land use types and measurement positions. We observed moderate to strong correlations between different plant parameters (e.g., LAI and chlorophyll or NDVI with *r* > 0.71; *p* < 0.001). Soil physical and chemical conditions also showed a strong to a very strong relationship in certain cases, such as total SOC to total N (*r* = 0.97; *p* < 0.001), SOC to sand content (*r* = 0.71; *p* = 0.001), or moderate and significant relationships such as GCI_S2_ and sand (*r* = 0.65; *p* = 0.003), LAI and silt (*r* = 0.59; *p* = 0.01), or NDVI and silt (*r* = 0.59; *p* = 0.01).

## Discussion

### Climatic conditions affecting soil and vegetation parameters

In the present research, we aimed at studying the connections between different environmental parameters and VIs while using data gathered from different land use types within proximity to each other. This study highlighted the distinct differences among the land use types examined. We also showed that differences between field and satellite measurements require careful interpretation. Our findings further support the initial hypothesis that cimate change related variations in environmental parameters have measurable effects on plant parameters, including soecific VIs. The study was conducted under a continental climate with Mediterrean influences; however, regional and microclimatic variability plays a critical role in shaping VI responses and spectral sensitivity to water and heat stress. Compared to generally cooler and more humid conditions, such as in northern countries, the warmer and often drier environment can amplify and accelerate stress-induced spectral responses. This can be particularly noted in the red and near-infrared regions through changes in canopy structure, pigment composition, and water content^39,40^. Consequently, the sensitivity and transferability of vegetation indices could be further interpreted in a climatic context. This highlights the importance of integrating findings across regions with differing environmental conditions. Several factors influence VIs and soil parameter changes, such as soil temperature, soil moisture, solar radiation, vegetation cover, or topography^41,42^. Rising soil temperature during the summer without significant precipitation amounts accelerates soil moisture loss, which increases plant stress. During our measurement time, the research sites experienced drought conditions, leading to observable changes in VIs related to plant stress during the growing season. The daily average air temperatures were higher than 20℃ for three consecutive months, which is higher than the research catchment’s long-term average^27^. We found that under elevated heat and low rainfall amounts, the soil moisture values across all three land use types dropped below the wilting point, limiting water availability for shallow-rooted plants and causing various visible symptoms of water stress for sunflower and grassland plants.

NDVI is one of the most accessible and widely utilized indicators of plant health, which, by monitoring vegetation status, is suitable for observing associated changes in soil moisture and temperature^43^. However, the point at which stress signals become detectable might be too late for timely management action^6^. Wong et al.^5^ found that NDVI can be invariant for short-term stress signals, while PRI might be a better option, which can be a more sensitive indicator of plant stress. The wide range of overall VI values in the grassland reflects the diversity of plants at the research site, hence vegetation type is an important factor for spectral data. Moreover, the complexity of plant physiology and their sensitivity to environmental factors can vary significantly among species. At our grassland site, the continuous decrease in PRI values over time indicated the long-term effects of water stress, similar to earlier studies^44,45^. In our data, PRI values showed a relatively linear decrease; however, a nonlinear response over time may also occur^46^, potentially leading to irreversible plant damage. Grassland NDVI, from both handheld and satellite sources, clearly reflected patterns of heat and water stress during mid-summer and showed a gradual increase following rainfall events. In contrast, forest NDVI remained relatively stable throughout the vegetation period, only changing in the fall as leaves naturally changed their color. Thus, NDVI might indicate drought for deep-rooted vegetation under long-term extreme conditions^12^. Therefore in special cases, PRI might be a better option for detecting plant stress. However, its use depends on the availability of hyperspectral images from satellites or UAVs.

### Relationships among soil and vegetation parameters

We found a clear, positive correlation between NDVI and SWC; however, our data mostly included low to medium SWC values, preventing us from investigating high soil moisture stress. Meteorological data from the last 15 years suggest low possibilities for high water stress at the study catchment^27,47^. A similar but opposite trend was observed for soil temperature; higher soil temperatures correlated with lower NDVI values, with the highest values observed for grassland. Similar findings were reported by Swain et al.^48^, where higher sensitivity to VIs change was noted for grassland compared to cropland. While these findings were generally expected, we observed that land use type significantly affected the strength of the connections between environmental parameters and VIs. The relationship between SWC and NDVI can vary among land use types. For example, Adegoke and Carleton^49^ reported stronger correlations between SWC and NDVI in forests than in croplands. In our study, we found similar connections (r = 0.73 or 0.71 for cropland and forest, respectively). The correlation values between SWC and NDVI data for individual land use types were stronger when considering all data combined, indicating that broad conclusions should be avoided and land use types or plant species should be studied separately. Conversely, the relationship between soil temperature and NDVI exhibited a consistent trend across all data and land use types. This finding indicates that soil temperature exerted a significant influence on vegetation growth and photosynthetic activity. Our results align with previous studies reporting negative relationships between land surface temperature and NDVI^50,51^. The high correlation values may be attributed to increased biomass production, which is associated with enhanced evapotranspiration and consequently lower soil temperatures^52^. This mechanism might be an important factor in our study, as the forest having the highest biomass, also exhibited the lowest soil temperatures, consistent with strong canopy shading and reduced direct sunlight. Our findings indicate that NDVI might be suitable for monitoring soil moisture and temperature changes through vegetation status. However, more advanced indicators, such as relative surface evapotranspiration index, temperature-vegetation dryness index, or temperature-soil moisture dryness index, may be effective in identifying water-stressed periods by capturing intricate environmental patterns^53^.

### Integrating remote sensing and field data for validation

Data from RS can help understand the spatial heterogeneity of the agricultural field, and can also be used for determining hotspots of concern. One key difference between crop-specific measurements from handheld instruments and satellite imagery is that satellite data include background signals, such as soil, ground cover, and surrounding vegetation. Discrepancies between in situ leaf or plant-scale measurements and satellite-derived values are largely attributable to areas not occupied by the target crop^12^, while fluctuations in vegetation cover rate make it difficult to define clear connections between VIs^54^. This was shown in our data: the canopy covers during the vegetation period were close to 100% for forest and grassland, while sunflower canopy cover increased sharply over time as plants reached maturity. Thus, discrepancies between RS and field data change throughout the stages of vegetation growth and development. Ferrara et al.^55^ found that the discrepancy between remotely sensed and in situ spring phenology metrics varied throughout the beginning of the growing season. Further, Younes et al.^56^ found that even differences in study areas can influence phenology, as larger areas may conceal species composition and plant growth stage details, making comparisons across sites of varying extent unreliable. This highlights the importance of careful spatial and temporal planning of field measurements.In our measurements, forest NDVI_S2_ values were higher compared to NDVI_F_, emphasizing that satellite sensors primarily capture the upper canopy, whereas handheld instruments measure vegetation at the leaf scale. Therefore, these methodological differences should be considered when substituting one approach for the other. Also, a minor temporal discrepancy between field measurements and satellite image acquisition may introduce additional uncertainty when comparing retrieved VIs such as NDVI. However, minimizing the temporal discrepancy between field measurements and satellite overpass can partially address this issue. Differences in solar angle and atmospheric conditions between field sampling and satellite overpass can affect the comparability between measured and S2 retrieved reflectance values^57^. In our study, however, field samplings were conducted within a short temporal window around the satellite acquisition date, and data were omitted when the meteorological conditions were not closely identical.

LAI and canopy cover have been shown to strongly correlate with multiple plant VIs^58–60^. We found that LAI can be a close proxy for estimating sunflower NDVI and GCI_S2_, as indicated by the high correlation coefficients (r = 0.98 and r = 0.96, respectively), based on 42 averaged data points. The relationships for grassland and forest were less pronounced (Fig. 6), regardless of plant growth stage. Despite the expected strong relationships between LAI and other VIs, the number of data points is critical for ensuring the reliability of these relationships. It is also important to note that extreme environmental conditions may require additional attention to measurement techniques or methods. During prolonged drought conditions, such as those experienced in July and August in this study, grassland biomass may decline to levels at which LAI measurements become difficult to obtain reliably and may require destructive sampling to achieve accurate estimates.

An increase in chlorophyll and nitrogen content in leaves can be observed under water-stressed conditions^61,62^. Although, a lack of change in leaf chlorophyll content might also be expected^63^, similar to the findings of our study. The average leaf chlorophyll value of the grassland was significantly lower than that of the cropland and forest. This can be attributed to the shallow topsoil in the grassland, e.g., plant roots have limited depth to develop, and the different plant species compete for available water. While VIs such as NDVI are highly influenced by LAI or canopy density, other S2-retrieved bands, such as the Structure Insensitive Pigment Index (SIPI), can help reduce the effect of structural variability^64^ and may be further used for monitoring the health and stress levels of plants. The time of the natural life cycles of different plants varies significantly. Sunflowers are already in the senescence period by the end of August; hence, sharp declines in NDVI and chlorophyll contents occur even in the absence of water and heat stress. However, several types of grass and plant species in grasslands can still have higher NDVI and chlorophyll contents during fall. It can happen after either adequate precipitation events or the more drought-tolerant nature of the plants can still function effectively. In general, our study highlighted that the timing of data collection at a given land-use site is critical, and comparisons among different plant types should account for temporal variability in their phenological stages.

### Soil-plant-water system and relations

Plant physiological traits, such as canopy cover percentage, affect rain interception and consequently, soil water content and soil water regime. In our study, grassland had the highest water content of the topsoil among land use types, while the forest showed significantly lower SWC values. This result was unexpected given the high vegetation cover and LAI of the forest, and the lower summer soil temperatures, anticipated to support lower evaporation rates at this site. However, the differences among soil parameters, namely the dense litter layer and the interception amount of rainfall in the forest, might outweigh the environmental parameters. The forest floor is covered with fallen leaves throughout the year, which enables smaller rain events to infiltrate the soil^65^. Hence, its additional interception value is high. After larger precipitation, this litter layer can help retain water and reduce soil moisture loss to evaporation, but in our research sites, larger precipitation events are getting rarer. In our study period, the long drought condition started after the end of June and lasted till mid-August, during which time all three land uses had very low SWC. For deep-rooted plants such as trees in forests, the roots can accelerate the soil moisture retrieval from lower soil layers^66^, further depleting SWC. The interception effect in our forest site was more pronounced after precipitation events at late-August. Canopy openness can further influence the spatial heterogeneity of SWC^67^, a pattern supported by our findings, as measuring points with lower LAI values exhibited lower overall SWC.

Soil physical and chemical properties, such as hydraulic conductivity, grain size distribution, and SOC contents, are influencing factors of soil water retention potentials and consequently VIs. Soil texture can greatly influence SWC, as higher clay content concurrent with higher aggregate stability often helps retain more water^6^. The significantly higher clay contents of the cropland and grassland compared to the forest soil could further enable higher soil moisture to be retained. However, it seemed to be less influential compared to the vegetation coverage percent or the LAI of the sites. One of the most important soil chemical factors influencing SWC, along with VIs, is the soil organic matter (SOM) or SOC content^68,69^. It was found that higher SOC content in soils can help retain more water^70^; however, in our study, the highest SOC was observed for the forest with significantly lower SWC. This finding further highlights that, in certain cases, the SWC can be more dependent on the throughfall rain amount rather than evaporation processes.

In the present paper, we observed several important results regarding the overall soil-plant-water system; however, the study covers a limited time interval, comprising only a single full vegetation period. Although the study year represents extreme drought conditions, extending the research to a multi-year approach would further enhance the robustness of our findings. Long-term studies could incorporate a broader range of vegetation indices, extended satellite time series, and varying climatic conditions at the same research sites, thereby enabling a more comprehensive understanding of the soil-vegetation processes.

## Conclusions

Our results highlight the complex interaction and interconnection between meteorological conditions, land use types, soil parameters, and plant physiological traits. Our study showed that land use type governs the strength and nature of interactions between environmental parameters and vegetation indices. Soil temperature exhibited a strong and consistent relationship with NDVI across all investigated land use types, underscoring its dominant role in regulating vegetation growth and photosynthetic activity. SWC can be used individually for each land use type; however, our findings are valid only under drought or near drought conditions, as the range of our data was restricted to medium SWC levels. Our study further demonstrated that LAI emerged as a reliable proxy for estimating NDVI and GCI in sunflower systems. Field-measured and satellite-retrieved NDVI values showed good correlations, especially with data gathered from the grassland. In the forest, dense litter layers and high rainfall interception exerted a stronger control on soil water dynamics than other environmental parameters, highlighting the importance of soil-vegetation processes specific to land use when interpreting remotely sensed vegetation signals.

This study raises several questions to be considered for future research directions. Three distinct land-use types were investigated, each representing a different intensity of anthropogenic influence. Forest ecosystems were characterized by minimal human disturbance, grasslands by moderate levels of interaction, and croplands by high levels of anthropogenic impact, all of which influence soil and plant properties over time. Although the findings contribute to a better understanding of how agricultural management practices affect soil-plant-water systems, the primary focus of the study was on plant physiological responses to water and heat stress, with comparatively less emphasis on soil variability or different climatic conditions. Therefore, future directions should include the potential of satellite-based composite indices that integrate climatic and soil properties as a promising avenue for improving comparability and scaling our findings under different land use and land cover.

## Data Availability

Current study data can be available upon request from the authors. For any data inquiries, please contact Agota Horel ( [horel.agota@atk.hun-ren.hu](mailto:horel.agota@atk.hun-ren.hu) ).
